# Breastfeeding experiences and perspectives on support among Chinese mothers separated from their hospitalized preterm infants: a qualitative study

**DOI:** 10.1186/s13006-019-0242-9

**Published:** 2019-11-01

**Authors:** Yuanyuan Yang, Debra Brandon, Hong Lu, Xiaomei Cong

**Affiliations:** 1 Peking University School of Nursing, 38 Xueyuan Road, Haidian District, Beijing, 100191 China; 2 Duke University School of Nursing, 307 Trent Drive, Durham, NC 27710 USA; 3 University of Connecticut School of Nursing, 231 Glenbrook Road, Storrs, CT 06269-4026 USA

**Keywords:** Breastfeeding, Infant, Premature, Experience, Support, Separation

## Abstract

**Background:**

Chinese mothers of preterm infants often face obstacles to breastfeeding and commonly experience prolonged maternal-infant separation when their high-risk infants are hospitalized in a Neonatal Intensive Care Unit (NICU). This separation hinders mother-infant attachment and the establishment of breastfeeding. Currently, little is known about Chinese mothers’ experiences breastfeeding their preterm infants, or their support needs. The aim of this study was to develop an understanding of mothers’ experiences breastfeeding a hospitalized preterm infant and the support needed to establish a milk supply during the period separation from their infants.

**Methods:**

A qualitative descriptive study was conducted in Beijing in 2017. A total of 11 Chinese mothers were individually interviewed while separated from their infants. The interviews were audio-recorded and transcribed verbatim. A thematic analysis involving a seven-step protocol identified key themes.

**Results:**

Mothers of preterm infants reported physically and mentally challenging breastfeeding experiences during the period they were separated from their babies. They viewed expressing breast milk as integral to their maternal role, even though some found expressing breastmilk exhausting. With little professional support available, the mothers depended upon nonprofessionals to establish breastfeeding.

**Conclusions:**

The study identified the difficulties mothers experienced establishing a milk supply while separated from their preterm infants, and the importance of access to health professional support.

## Background

A report released by the World Health Organization (WHO) identified that more than 15 million preterm infants are born each year. China has the second largest number of preterm births with 1.17 million born annually with those at high risk of adverse outcomes hospitalized in Neonatal Intensive Care Units (NICU) immediately after birth [1]. To avoid the effects of mother/infant separation, parents in many countries are encouraged to stay with their pre-term infants until they are discharged from hospital. While family integrated care is recommended and implemented in neonatal units world-wide, neonatal care policies vary from country to country [2].

In China, NICU facilities were first established in the 1980s. Currently many units do not allow parental visits or participation in caregiving, including breastfeeding. Consequently, Chinese pre-term infants frequently experience a prolonged period of separation from their mothers. This separation hinders both mother-infant attachment and the establishment of the mother’s milk supply [3, 4]. Poor emotional bonding and maternal concern for their infant’s health exposes mothers to stress, anxiety, depression and other negative emotions [5]. Chinese mothers separated from their infants, commonly experience postpartum anxiety and depression which can interfere with the establishment and maintenance of breastfeeding [6, 7]. Indeed, the greater the maternal stress, the more likely a mother will stop providing breast milk for her infant. Conversely, allowing mothers to stay with their preterm would encourage earlier exclusive breastfeeding [8].

Currently mothers separated from their pre-term infants face significant challenges such as inadequate milk supply, breast engorgement, delayed initiation of their milk supply and breastfeeding failure [9, 10]. Those who wish to breastfeed have to express breast milk until their infants are discharged from the neo-natal unit [11]. During the baby’s hospitalization most mothers struggle to establish and maintain an adequate milk supply as hand and breast pump expression are not as effective in stimulating and maintaining a milk supply as suckling an infant at the breast [12]. Consequently, the separation of mothers from their babies while being cared for in Chinese neonatal units is a significant impediment to the initiation and maintenance of breastfeeding.

In a previous longitudinal observational study, we identified only 22.5% of preterm infants were exclusively breastfed at 6 months of age, and that mother-infant separation was a contributing factor to the low rates of exclusive breastfeeding in this country [6]. To date, the immediate impact of post-natal maternal-infant separation on Chinese mothers’ experience of establishing breastfeeding remain largely unknown and in need of further investigation.

In view of the challenges faced by mothers of pre-term infants, it is essential to provide breastfeeding support. Studies in other countries demonstrate that mothers value support received from healthcare professionals, and associate adequate provider support with better breastfeeding outcomes [13]. In China, healthcare providers’ knowledge on breastfeeding preterm infants is limited [14]. A multi-center study identified that only 2 of 15 Chinese NICUs representing the highest level of neonatal care chose breast milk to feed very low birth weight preterm infants and clinical practices breastfeeding preterm infants was inconsistent between districts [15]. With no national evidence-based clinical guidelines for breastfeeding preterm infants, a mother’s access to adequate and appropriate breastfeeding support from health professionals is compromised and a better understanding of their experience is needed. Accordingly, this study sought to understand Chinese mothers’ experiences of expressing breast milk for their pre-term hospitalized infants and to identify the support they needed to establish and maintain an adequate milk supply.

## Methods

### Participant characteristics and study design

A total of 11 mothers of preterm infants admitted to three level III Beijing NICUs were recruited in 2017. All three Units encouraged mothers to express and transport milk to the hospitals which lacked milk banking facilities, did not provide hospital-grade breast pumps, or allow mothers to stay with their infants. One Unit only permitted weekly maternal visits of 10 min duration!

The mothers recruited for the study were aged over 18 years, spoke Mandarin Chinese and had no contraindications to breastfeeding. Their infants were born normal and healthy at less than 37 weeks gestation. The study employed Husserl’s descriptive phenomenological approach, that is, one which attempts to describe the experience of people without interpretation. This approach involves direct exploration, analysis and narration of specific phenomena through setting aside existing presumptions [16].

The research team employed semi-structured, individual interviews to describe the mothers’ breastfeeding experiences while separated from their infants. The original version of the interview guide was scrutinized by a pediatric nursing expert and preterm infant care specialist to confirm the suitability of the questions. The interview guide was pre-tested on one mother to ensure acceptability of the questions. The pre-test data was not included in the study. Culturally, the word “breastfeeding” in Chinese means feeding the infant directly at the breast, as well as providing expressed breast milk. The following questions were asked in order:

1. What were your experiences and feelings of breastfeeding your preterm infant/s?
2. How did you cope with the difficulties or problems when breastfeeding your infant/s?
3. What do you think of the support on breastfeeding you have received?

### Data collection

The study was approved by Peking University Institutional Review Board. In order to  ensure we covered a wide range of breastfeeding experience, we employed purposeful sampling to recruit mothers for the study. Characteristics considered for recruitment included education levels, geographic residence and feeding patterns. The sample size was determined by data saturation [17]. That is, data collection ceased once no new information was obtained.

Potential participants were selected via a review of the medical records of preterm infants. Mothers who met the inclusion criteria were approached, informed of the study purpose and significance by the head nurse of each NICU, and verbal consent was obtained. To avoid coercion, mothers were informed of their right to decline to participate in and/or to withdraw from the study and assured such a decision would not affect the care of their infants. They were also assured that the interviewer was not employed as a nurse in their infant’s NICU and confidentiality over data collected was guaranteed.

The time and location for each interview was scheduled by the head nurse in consultation with each mother. All the interviews were conducted at the hospital by the first author prior to the infant’s discharge. The head nurse introduced the researcher at the beginning of the interview, a consent form was signed and a brief demographic, obstetric and breastfeeding questionnaire completed.

Interviews were conducted in a quiet room separate from the NICU to avoid interruptions. Only the mother and the interviewer were present during the approximately 30 to 45 min interviews. Each interview was audio recorded and the mother’s nonverbal behavior such as expressions and movements were simultaneously documented in the interviewer’s field notes. To encourage mothers to articulate their experiences and feelings, the interviewer employed counselling skills such as listening, paraphrasing and expressions of empathy [18].

### Data analysis

Following transcription of each interview, analysis of the qualitative data analysis was initiated. To ensure reliability, the results were independently scrutinized by two researchers. Key themes were identified collaboratively employing Colaizzi’s phenomenological analysis [19]. This process focused on thoroughly understanding the meaning of responses through repeated reading of the interview transcripts. Memos recorded preliminary ideas and assumptions, recurring ideas were coded, and the themes refined through checking, comparing and discussing the findings among the research team. During the data analysis and interpretation, the team attempted to suspend any prior knowledge or bias in order to grasp the experiences and feelings of participants [16]. A bilingual researcher verified the accuracy of the translated transcripts.

## Results

The characteristics of the participants are shown in Table 1. Most of the participants were primiparous (7/11) and had given birth by Caesarean section (7/11).

**Table 1:** Participant characteristics (*N* = 11 mothers)

Characteristics	Frequency (n)
Primiparous	7
Twin gestations	4
Mode of birth	
Caesarean section	7
Vaginal delivery	4
Feeding type at discharge	
Exclusive breastfeeding	6
^a^Partial breastfeeding	4
Exclusive formula	1
Maternal education	
Associate degree	1
Bachelor	6
Master	4

^a^Partial breastfeeding refers to breast milk combined solid or semi-solid foods, formula or non-human milk

### Themes

#### Theme I: providing breast milk is a way to identify as a mother

With their preterm infants hospitalized immediately after birth and limited access to their infants, the mothers experienced difficulties as they attempted to establish their maternal role. *“I haven’t seen my [baby] ‘till now* [from birth to discharge] *… I don’t feel I am a mother.”*, *“There is no real sense of motherhood.”* A significant source of the mothers’ anxiety was their concern for the health and safety of their infants. One admitted that *“When the child is hospitalized, I feel very anxious”*, while another described the situation as *“very stressful.”* Adding to maternal anxiety was their limited milk supply, indicated through comments such as *“I was anxious when I had no milk after delivery*.”

The mothers also reported a sense of helplessness over their inability to participate in their infant’s feeding and care. One reflected, *“I don’t know how I got through those days, what I hoped [for] every day was the doctor’s call.”* Others reported that the physicians only telephoned the parents if the baby’s condition improved or worsened; if the improvement or deterioration was mild, the call was only made if the physicians had time or felt an update was necessary.

Denied their maternal role, the women admitted they did not feel like they were mothers. “*It’s the breasts starting to go up and milk oozing out that let me think I am a mother*”. All believed that providing breast milk was the only way to establish a direct connection with their infant. As one observed, “*Preterm infants are more fragile than full-term infants. The only thing mothers can do is to express more milk for them*”. This observation was endorsed by another who admitted, *“When I realized my milk could help him get well sooner, I felt better*.”

#### Theme II: perceptions and intentions

While the mothers felt that providing breast milk for their infants was part of their maternal role, six indicated either mild or no intention of expressing breast milk. One, for example, was “… *not sure I can keep on breastfeeding for a long time*”, while another “… *didn’t want to breastfeed”.* Several factors contributed to their ambivalent attitude towards breastfeeding. Some misunderstood the nutrition of breast milk through comments such as: “*I don’t think breast milk can provide enough nutrition for preterm infants. They need large amounts of nutrients to get the weight gain. But the breast milk looks pretty dilute and the color is clear. It seems that breast milk has fewer nutrients than the formula.*” “*Both the amount and quality of breast milk are not good enough. The child will grow slowly if she only [gets] breast milk.*” “*I think that formula is more nutritious for the [baby’s] weight gain … breast milk is definitely not as good as formula*”. Others questioned the impact of their diet on the benefits of their breast milk: “*I am afraid that the quality of my milk is not good, and the amount is inadequate… due to my wrong diet, which will affect the health of my kid*”.

The opinions and behavior of people in the community also affected the mothers’ resolve to breastfeed. Some reported *“… a lot of my friends and colleagues don’t breastfeed.”* Others observed, *“My siblings fed their children with formula*”. *“Formula is also very good for the child’s development*”. “*My parents asked me not to breastfeed my baby because formula feeding is easier than breastfeeding”.* Negative experiences breastfeeding an older child also influenced a mother’s intention to breastfeed, with one admitting that “*When my first kid was born, I [wanted] to breastfeed. However, I didn’t have enough milk… [and breastfeeding] … caused me fatty* [weight gain]*. [So] I’m not insisting on it now.*”

A preterm birth poses a threat to the health of both the mother and the infant. Hospitalized preterm infants are vulnerable to complications and even life-threatening events after birth. Consequently, all the mothers were more concerned over their infant’s health than establishing breastfeeding. Some preterm births are caused by maternal diseases such as placenta previa, uterine adenomyosis, hypothyroidism, and diabetes. Most participants experienced multiple problems during pregnancy and childbirth, with the majority indicating an ambivalence attitude towards breastfeeding. One mother, for example, argued “… *formula feeding is much easier for the mother; I don’t want to be so tired”*; another didn’t want to breastfeed *“… because I have already suffered a lot during pregnancy”*; and a third admitted that *“ … as long as the child is discharged from the hospital, whatever he eats does not matter.*”

Two mothers were concerned over the negative impact of breastfeeding would have on their lives, with one admitting *“… because of my work, I don’t want to breastfeed.”* Others worried about changes in body shape associated with breastfeeding. As one observed “… *it is not easy to ensure adequate milk for the kids without getting fatty* [gaining weight]*.”* Another was concerned that breastfeeding *“… may cause… sagging of the breasts.*”

#### Theme III Milk expression makes mothers exhausted

Most mothers reported difficulty expressing milk, with one describing the feeling as experiencing *“…*a nervous breakdown*”* and another as *“… uncomfortable* [as] *the areola was hurt and painful*.*”* Most found frequent expression challenging with one reporting she was *“… too tired at night. No matter how late at night, as long as the alarm clock sounds, I have to sit up to express milk …* [it] *needs perseverance*.” Others “… *almost give up milk expression because of … mastitis.”*

#### Theme IV: health professional support on breastfeeding are urgently needed

##### Health professional support

All the mothers had confidence in information they received from health providers. “*Of course, I believe the healthcare providers. They are the experts.” “I trust doctors and nurses.*” Although they preferred health professional support, they recognized hospital staff were busy and had limited time to offer any support. Their realization that *“… doctors and nurses are too busy…* [and] *one-on-one guidance is not feasible”* prompted calls for group or mother-directed education. As one mother observed, *“Classes provided by the health professionals, like a prenatal school for pregnant women… [would be] convenient for both providers and parents”.* Another felt that “*videos made by the hospitals…* [would be] *more useful and reliable than the lay person* [as health professionals] *can… correct the wrong ideas of the elderly members of my family and avoid the quarrels between us about feeding my baby.*” Others indicated that “*professional books published by doctors and nurses would be very helpful,*” observing that an “*official booklet is more convincing than... hearsay.*”

##### Dissatisfaction with non-professional support

As indicated above, the mothers received limited support for establishing their milk supply and coping with the breastfeeding problems. Some reported receiving helpful information from nurses following delivery; another noted that while “… i*n the maternity ward, the nurse asked me to watch a video about newborn care. It was very helpful.”* A pediatric nurse urged another mother *“… to express more frequently to increase the milk supply.”* Lacking access to professional support some turned to alternative sources of support and information. *“… there was a hard mass on my breast. I later found a Cui Ru Shi* [unlicensed lactation consultant] *to massage my breast to help* [the engorgement] *subside.*” More than half the mothers consistently sought help through social media sites. One reported *“I asked for help in a WeChat group* [a social media group] *which is comprised of many mothers*”; others “*bought parenting books*” or consulted other mothers and colleagues who had experienced similar problems with breastfeeding or milk expression. Web-based resources were also commonly used. “*I often search the internet and there are some parenting websites.*” “*Milk expression techniques are all learned online.”* “*I have been exploring* [the internet] *by myself*.”

Regardless of the source of breastfeeding support, there were occasions when the mothers could not obtain the information they needed. “*There are many people in the WeChat group, but no one answered my questions, unlike one-on-one guidance where you will definitely get an answer.*” “*I haven’t found a book …on breastfeeding for preterm infants.*” They were also reluctant to seek advice from the doctors and nurses as they felt they were *“… too busy to answer questions* [and were] *ashamed to increase the burden on them*”. Some reported feeling confused by contradictory opinions received from different information sources. *“My husband and I once argued. We finally found that we got the ideas from different books. We* [didn’t] *know which statement is right. It* [was] *confusing.”* Others were troubled by unsolicited support with one noting that *“Other mothers’ advice is not completely suitable for my kid*.*”* Generally, the mothers were dissatisfied and frustrated over the support they received from non-professional sources.

## Discussion

Expressed breast milk or breastfeeding provides not only essential nutrients for preterm infants but also helps establish emotional connections between mothers and their infants [20]. This study found that when mothers are separated from and unable to care for their infants, they do not believe they are fulfilling their maternal role. This finding is consistent with an earlier study [21].

Our findings also demonstrate that while mothers recognized that providing their hospitalized infants with breast milk made them feel more like a mother, the challenges they experienced expressing breast milk and establishing breastfeed were extremely frustrating. Similar findings have been reported in other studies on low birth weight and late preterm infants conducted outside China [5, 11, 21]. Frequent breast milk expression can lead to physical exhaustion and breast pain. Consequently, mothers expressing breast milk are at higher risk of inadequate milk supply than those feeding from the breast [22]. This knowledge contributes to mothers’ anxiety that they will be unable to provide sufficient milk for their infants. Furthermore, hospitalized preterm infants, particularly those of small gestational age, are often unstable, adding to a mother’s distress while separated from her infant. A combination of these factors contributed to the negative emotions the mothers experienced while separated from their preterm infants. Consequently, it is important that a mother’s emotional experience if separated from her infant after delivery is taken into consideration by health professional and hospital policies. Further research is needed to identify effective interventions that emotionally support mothers of hospitalized pre-term infants.

Research suggests that mother-infant separation, difficulties with milk expression and concerns over inadequate milk supply inhibit the provision of breast milk for their hospitalized preterm infants [23]. When separated from their infants, the only strategy available to breastfeeding mothers is manual expression. The difficulty is that milk expression alone is often insufficient to establish and maintain an adequate milk supply. Skin-to-skin kangaroo care and early infant sucking at the breast are recommended as effective strategies to establish breastfeeding among preterm infants [13]. Through allowing mothers to stay with their preterm infants in NICU, the establishment of exclusive breastfeeding would be promoted [8]. Consequently, there is a need for policies in Chinese NICUs that allow and encourage mothers and preterm infants to remain together and promotes kangaroo care to establish breastfeeding and improve mother-infant emotional closeness, health and wellbeing.

Although the mothers in this study believed providing breast milk was the only way they could support their hospitalized infants, they lacked the confidence and determination to continue providing expressed milk. Previous studies have demonstrated that mothers’ attitudes are important. Those with positive attitudes and clear breastfeeding goals are more likely to provide breastmilk for hospitalized infants and to breastfeed for longer [24, 25]. The low intention to breastfeed and skepticism over the value of breastfeeding among the mothers in this study was indicative of their lack of knowledge and confidence in their ability to breastfeed, and access to breastfeeding education and support [26, 27]. Moreover, the mothers’ intention to provide breast milk for their pre-term infants was influenced by colleagues, friends, and relatives [28–30]. In a pro-breastfeeding community, mothers are less likely to choose to formula feed. Consequently, there is also a need for community promotion and education to foster social environment that values the benefits of breastfeeding.

Our study demonstrates that a mother’s experience plays a pivotal role in the establishment and duration of breastfeeding. Studies show that mothers who have a successful breastfeeding experience are more likely to breastfeed subsequent infants for longer [31]. The need to return to work was a common cause of early cessation of breastfeeding or inability to exclusively breastfeed [32]. Almost all mothers the interviewed had a bachelor’s degree or above, and attached importance to their career development and personal image. Consequently, concern over the adverse impact of breastfeeding on their lives was an factor in the decision to provide breast milk.

Paternal support, peer support, healthcare provider attitudes and education can improve mothers’ confidence, skills and ability to breastfeed their infants skills and confidence [33–36]. In our study, mothers reported that they preferred the support offered by health professionals to express milk and breastfeed their infants. The limited availability of health professionals meant mothers did not receive the support they needed to establish and maintain their milk supply. Similar findings have been identified elsewhere [37]. In view of the lack of health professional support available to the mothers in this study, it was hardly surprising that they preferred publicly available information on breastfeeding such as videos. Consequently, there is an urgent need to re-configure healthcare education and support for breastfeeding mothers of preterm infants in China. This support requires training adequate numbers of lactation consultants and creating specialized, cooperative preterm infant care teams to support their mothers and families. There is also an urgent need for the establishment of clinical guidelines on supporting mothers of preterm infants to establish breastfeeding.

### Limitations

A major limitation of this study was the small number of participant interviews. The Chinese tradition of ‘sitting the month’, when a new mother is encouraged to remain sequestered for at least 1 month following childbirth to restore balance to the body hindered the researchers’ access to new mothers, suggesting telephone interviews maybe more effective when undertaking further research. Additionally, the employment of semi-structured interviews restricted the researchers’ ability to elicit rich data from the small number of participants. A further weakness was that most participants were recruited from urban areas and were well-educated. Consequently, the findings do not represent the experience of mothers from rural areas or those with lower levels of education. Further research on mothers drawn from a wider socio-demographic sample is needed to confirm the findings of this study.

## Conclusion

This study was limited to mothers who experienced long-term separation from their preterm infants. The key finding was that mothers faced a complex, negative experience when attempting to provide expressed breast milk for their preterm infants. Despite recognizing that producing breast milk supported the development of their maternal role, some harbored inaccurate perceptions of the value of breast milk, and benefits of breastfeeding for maternal health. Those who sought information on breastfeeding primarily relied on non-professional sources even though they recognized that health professional support was more desirable. Finally, the small sample size and use of semi-structured interviews limited the insight obtained on mothers breastfeeding hospitalized preterm infants in China. Further research is required to identify the most effective strategies to enable Chinese health professionals to support mothers to breastfeed their hospitalized preterm infants.

## Data Availability

The datasets analyzed during the current study are available from the corresponding author on reasonable request.

## Abbreviations

NICU: Neonatal Intensive Care Unit
VLBW: Very low birth weight
